# Development of a droplet digital PCR method for detection of porcine circovirus 4

**DOI:** 10.1186/s12917-023-03690-5

**Published:** 2023-08-22

**Authors:** Yangkun Liu, Xinru Zhang, Xueying Han, Jiaxing Liu, Lunguang Yao

**Affiliations:** 1 Henan Provincal Engineering and Technology Center of Health Products for Livestock and Poultry, School of Life Science and Agricultural Engineering, Nanyang Normal University, Nanyang, 473061 Henan China; 2 College of Veterinary Medicine, Northwest A&F University, Yangling, 712100 Shaanxi China

**Keywords:** Porcine circovirus 4, Droplet digital PCR, Real-time quantitative PCR

## Abstract

**Background:**

Porcine circovirus 4 (PCV4), a newly emerging virus that was first discovered in 2019, may pose a potential threat to the pig industry. Droplet digital PCR (ddPCR) is an absolute quantitative method that has high sensitivity and accuracy. In this study, we developed a novel ddPCR assay to detect PCV4. Furthermore, we evaluated the detection limit, sensitivity, specificity and reproducibility of the ddPCR and TaqMan real-time quantitative PCR (qPCR) and tested 160 clinical samples to compare the detection rate of the two methods.

**Results:**

The detection limit for ddPCR was 0.54 copies/µL, 10.6 times greater sensitivity than qPCR. Both ddPCR and qPCR assays exhibited good linearity and repeatability, and the established ddPCR method was highly specific for PCV4. The results of clinical sample testing showed that the positivity rate of ddPCR (5.6%) was higher than that of qPCR (4.4%).

**Conclusions:**

This study successfully developed a sensitive, specific and repeatable ddPCR assay for PCV4 detection, which can be widely used in clinical diagnosis of PCV4 infections.

**Supplementary Information:**

The online version contains supplementary material available at 10.1186/s12917-023-03690-5.

## Background

Porcine circoviruses (PCVs) are non-enveloped, circular single-stranded DNA viruses, which belong to the family *Circoviridae*, genus *Circovirus*. Until 2019, only three types of PCVs have been characterized, named PCV1, PCV2, and PCV3. PCV1 is a non-pathogenic virus derived from the porcine kidney cell line PK-15 [1]. PCV2 is confirmed to be the primary causative agent of porcine circovirus-associated diseases (PCVAD), resulting in huge economic losses to the global pig industry [2]. PCV3 was first identified in sows with porcine dermatitis and nephropathy syndrome (PDNS)-like clinical signs in 2015 in the United States and then in different countries around the world [3].

In 2019, a distinct novel PCV, designated PCV4, was discovered in the Hunan province of China in pigs with PDNS, respiratory and enteric signs [4]. Subsequently, PCV4 infections have been reported in Jiangsu, Anhui, Henan, Shanxi, Inner Mongolia and other provinces of China [5–8], as well as in South Korea [9], which indicate that PCV4 has a widespread epidemic trend and may be a potential threat to the global pig industry. To better investigate the epidemiology of PCV4, several diagnostic methods, including conventional PCR (PCR) [8], loop-mediated isothermal amplification (LAMP) [9], multienzyme isothermal rapid amplification (MIRA) [10], real-time quantitative PCR (qPCR) [6, 11] and enzyme-linked immunosorbent assay (ELISA) [5], have been developed for the detection of PCV4 infection. However, these methods may lack specificity and sensitivity, or do not allow direct quantification of viral DNA, thus rendering them unsuitable for routine diagnosis in the early stages of viral infection. Therefore, the development of a rapid, simple, and reliable diagnostic method is imperative for managing PCV4.

Droplet digital PCR (ddPCR) is an innovative third-generation PCR technology for absolute quantification of nucleic acids without the requirement of a standard curve [12]. The ddPCR uses the same target-specific primers and fluorescent probe as TaqMan-based qPCR. In ddPCR, the reaction mixture is partitioned into tens of thousands to millions water-in-oil droplets prior to massive parallel PCR amplification. At end point, each droplet is classified as positive or negative based on the recorded fluorescence signal, and the positive fraction of counted droplets is employed to calculate the target copy number using Poisson algorithms [13, 14]. The ddPCR method has been demonstrated to have higher sensitivity and specificity than qPCR, especially when the quantity of the target is very low [15–17]. At present, ddPCR has been used widely in the detection and quantification of a range of microorganisms, including other circoviruses, such as porcine circovirus type 2 (PCV2) [18], porcine circovirus type 3 (PCV3) [19] and pigeon circovirus (PiCV) [20]. However, no ddPCR assay is currently available for PCV4. In this study, a ddPCR assay was developed for detection and quantification of PCV4 in serum samples of pig. Furthermore, the sensitivity, specificity and repeatability of the PCV4 ddPCR assay was compared with qPCR.

## Materials and methods

### Plasmids, viruses and field samples

To prepare the standard positive control, the whole genome of PCV4-LY2020 (Accession no. MW759026) was synthesized and cloned into pBluescript II SK (+) vector. The recombinant plasmid named pSK-PCV4 was transformed into in *E. coli* DH5α and subsequently purified with a Plasmid MiniPrep Kit (OMEGA Biotech, Shanghai, China). The DNA concentration of the plasmid construct pSK-PCV4 was quantified using the NanoDrop 2000 spectrophotometer (Thermo Fisher, Waltham, MA, USA). The estimated copy number of the pSK-PCV4 plasmid in solution was calculated using methods described previously [21].

Classical swine fever live vaccine (CSFV, strain CVCC AV1412), and porcine reproductive and respiratory syndrome live vaccine (PRRSV, JXA1-R strain) were purchased from Wuhan Keqian Biology Co., Ltd. and stored in our lab. Porcine epidemic diarrhoea virus (PEDV) LYL strain (Accession no. ON960076), porcine rotavirus (PoRV) CC0812 strain (Accession no. JF835112), PCV2 SH strain (Accession no. HM038027), and porcine circovirus type 3 (PCV3; Accession no. MZ449239) culture media were stored in our laboratory. One hundred and sixty samples (105 blood samples, 55 tissue samples) were collected from 24 pig herds located in five cities (Nanyang, Zhengzhou, Pingdingshan, Xinyang and Luoyang) of Henan province from September 2019 to July 2022. Informed consent from the herd´s owners have been obtained to collect the samples used in this study.

### Primers and probe for PCV4 droplet digital PCR

According to the genomic sequence of the PCV4 strains listed in GenBank, the conserved sequences of PCV4 ORF2 gene was analyzed using MEGA 6.0 software. One probe and a pair of specific primers were prepared subsequently. The primer and probe sequences were as follows: PCV4-F (5’- CGTTCCAAGAGGGCGTG − 3’), PCV4-R (5’-GCCAGTAGGCGGAGATACC-3’), and PCV4-P (FAM-5’- ACCTCCC.

TCATGAAGCGCGCA-3’-BHQ1). All primers and probes were synthesized by Sangon Biotech (Shanghai, China).

### Nucleic acid extraction and reverse transcription

Viral RNA from PEDV, PoRV, PRRSV and CSFV were extracted using the RNA Viral Genome Extraction Kit (Solarbio Biotech Co., Ltd., Beijing, China), following the instructions of the manufacturer. Each viral RNA was employed for the synthesis of the first strand cDNA in a 20 µL reverse transcription (RT) reaction mixture containing 1 µg of total RNA, 4 µL of 5 × AMV buffer, 2 µL of dNTPs (2.5 mmol/L), 0.5 µL of RNase Inhibitor (40 U/µL), 1 µL of random primer, 1 µL of AMV reverse transcriptase (5 U/µL), and RNase-free­H_2_O and then incubated at 42 °C for 60 min and 95 °C for 5 min (Sigma-Aldrich, St. Louis, MO, USA). Viral DNA was extracted from PCV2 and PCV3 and using a DNA Viral Genome Extraction Kit (Solarbio, Beijing, China).

### Droplet digital PCR (ddPCR) assay

The ddPCR assay for PCV4 was performed in a TD-1 Droplet Digital PCR system (TargetingOne, Beijing, China) following manufacturer’s instructions. The reaction volume was 20 µL, containing 10 µL of 2 × ddPCR Supermix (TargetingOne, 23,003), 800 nM of each primer PCV4-F/R, 250 nM of PCV4-P probe, and 2 µL of the template. The reaction mixture and 180 µL oil were placed in a droplet generator, followed by heat-sealing for PCR. In order to optimize the annealing temperature, the amplification reaction protocol was as follows: 95 °C for 10 min, 40 cycles at 94 °C for 30 s and a temperature gradient from 55 to 61 °C for 1 min; the temperature ramp rate was set to 1.5 °C/s on a T100 thermal cycler (TargetingOne, Beijing, China). Finally, the droplets were analyzed using a chip reader (TargetingOne, Beijing, China). Then, ddPCR was optimized for primer and probe concentrations (300:200 nm, 800:250 nm, 500:300 nm and 400:400 nm). The ddPCR was performed in triplicate.

### qPCR assay

The qPCR assay for PCV4 was performed with the same primers and probe as ddPCR. The PCR was performed in 20 µL volume, including 10 µL of 2 × TaqMan™ Fast Advanced Master Mix (Thermo Scientific, Waltham, MA, USA), 1.6 µL of each primer (10 µM), 0.5 µL of probe (10 µM), 2 µL of the template and 4.3 µL of ddH_2_O. The PCR was conducted as follows: 50 °C for 2 min, 95 °C for 2 min, 40 cycles at 95 °C for 20 s and 57 °C for 20 s.

### Analytical sensitivity and repeatability

Dilutions of pSK-PCV4 plasmid ranging from calculated 2.0 × 10^5^ to 2.0 × 10^1^ copies per µL were used in analytical sensitivity determination of the ddPCR and qPCR assays. Two microliter of each plasmid dilution was used as template to ascertain the detection limit (LoD), which represents as the highest dilution detected by each PCR assay. To ensure the assay result accuracy, inter-assay and intra-assay repeatability tests were performed three times independently.

### Analytical specificity

To investigate specificity, assays including PCV4, PCV3, PCV2, CSFV, PEDV, PoRV, and PRRSV as templates were evaluated. Nuclease-free water was used in place of samples as no template control.

### Clinical sample detection by qPCR and ddPCR assays

To evaluate the applicability of the method, a total of 160 clinical samples from the pigs without any symptoms were assayed using the above ddPCR and qPCR procedure in parallel. In qPCR, any sample that has a Ct value more than 40 was considered as negative. Samples giving inconsistent PCR results were further verified by sequencing.

## Results

### Development of a PCV4 ddPCR assay

**Fig. 1:**
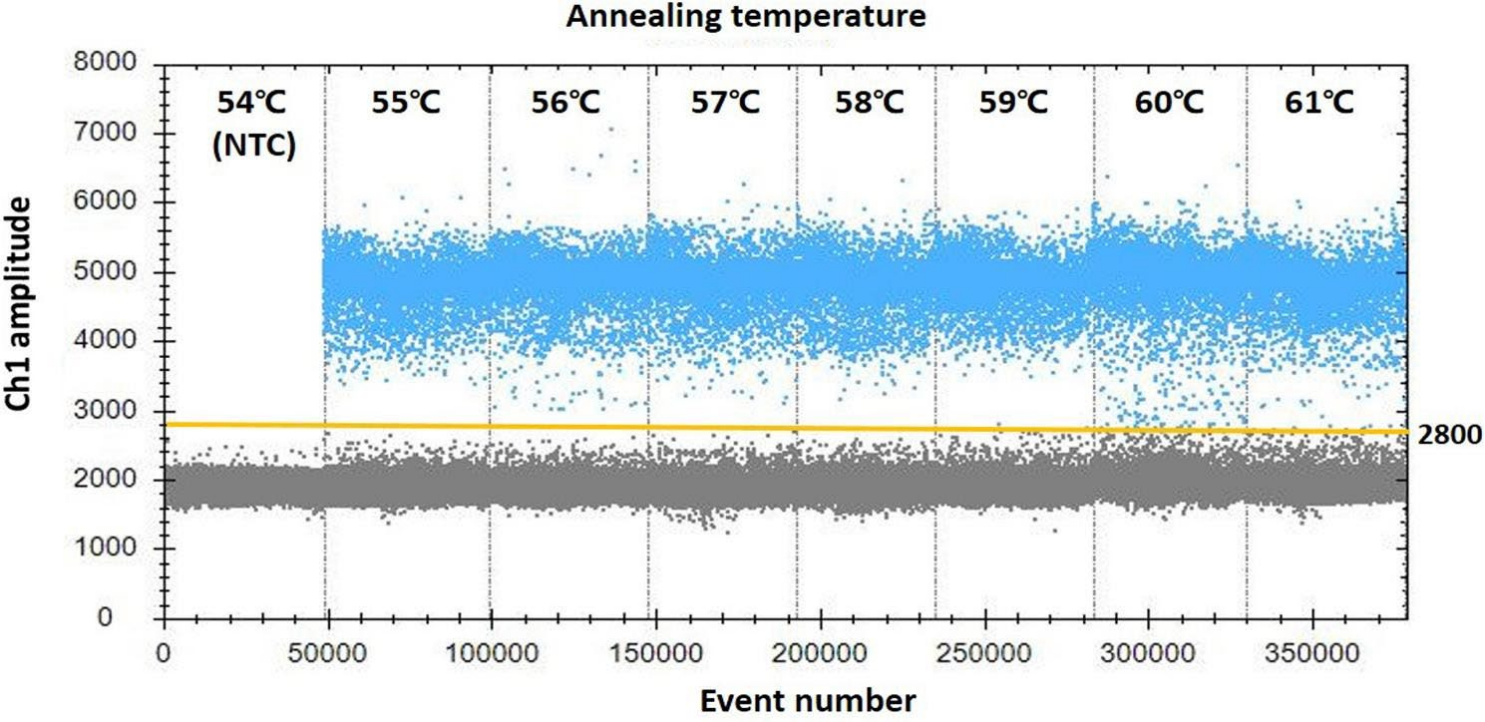
Influence of annealing temperature on the porcine circovirus 4 ddPCR. The assay was conducted across an annealing temperature gradient: 54, 55, 56, 57, 58, 59, 60 and 61℃. NTC = no template control. The blue dots are positive droplets, and the grey dots are negative droplets. The manually set threshold for droplet positivity is represented by the yellow horizontal line. 59℃ was chosen as the optimal annealing temperature in further assays

**Fig. 2:**
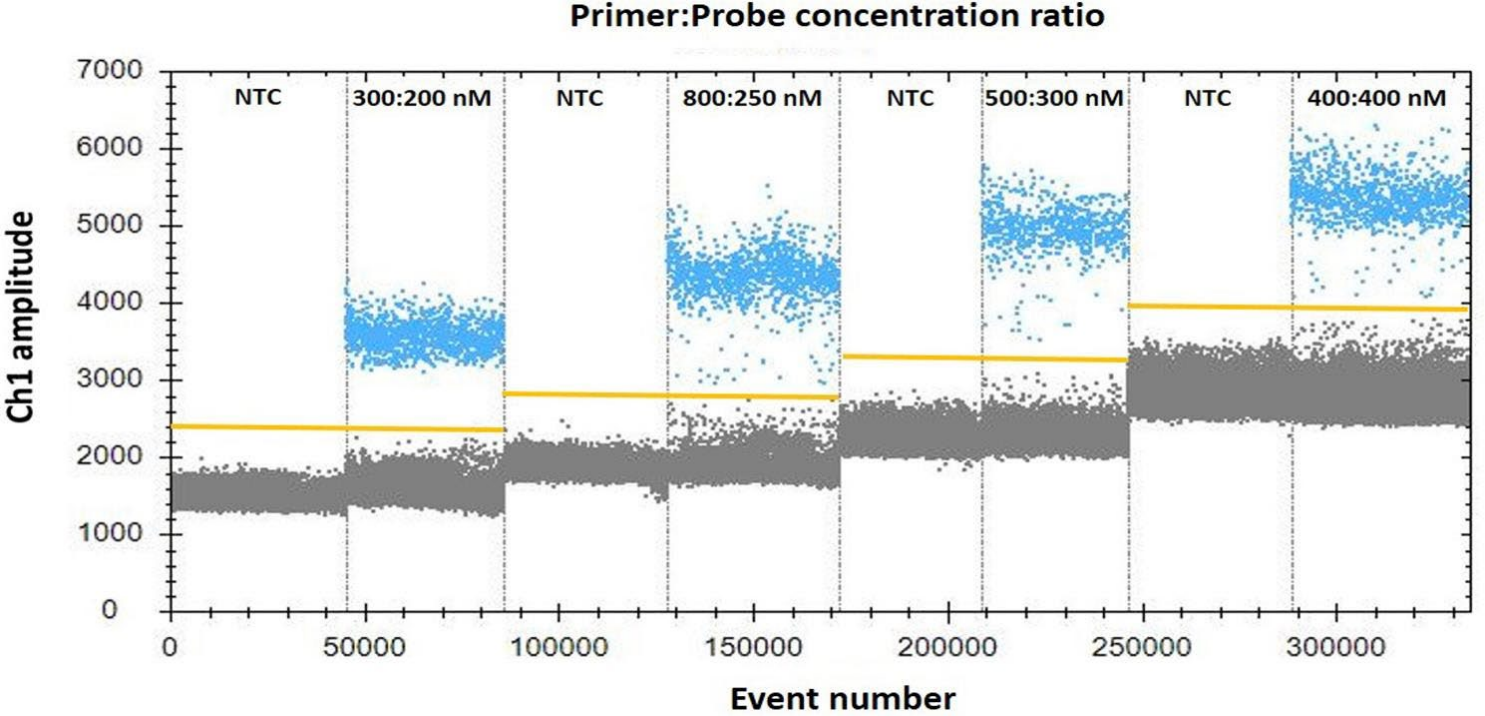
Influence of primer-to-probe concentration ratio on the porcine circovirus 4 ddPCR system. The assay was conducted under different primer and probe concentration ratios: 300:200, 800:250, 500:300, and 400:400. NTC = no template control. The blue dots are positive droplets, and the grey dots are negative droplets. The manually set threshold for droplet positivity is represented by the yellow horizontal line. 300:200 nm was chosen as the optimal primer-to-probe concentration in further assays

For ddPCR, annealing temperature gradients from 55 to 61 °C were performed to optimize the separation between positive and negative partitions. The results indicated that 59 °C provided the greatest difference in the fluorescence signal between the positive and negative droplet populations (Fig. 1), thus it was chosen as the optimal annealing temperature. To further determine if the ddPCR system for PCV4 could be improved, the primer-to-probe concentration was optimized. The results suggested that the optimal concentration ratio was 300:200 nM because this ratio of reagents resulted in optimal separation between positive and negative droplet populations (Fig. 2).

### Analytical sensitivity and reproducibility

**Fig. 3:**
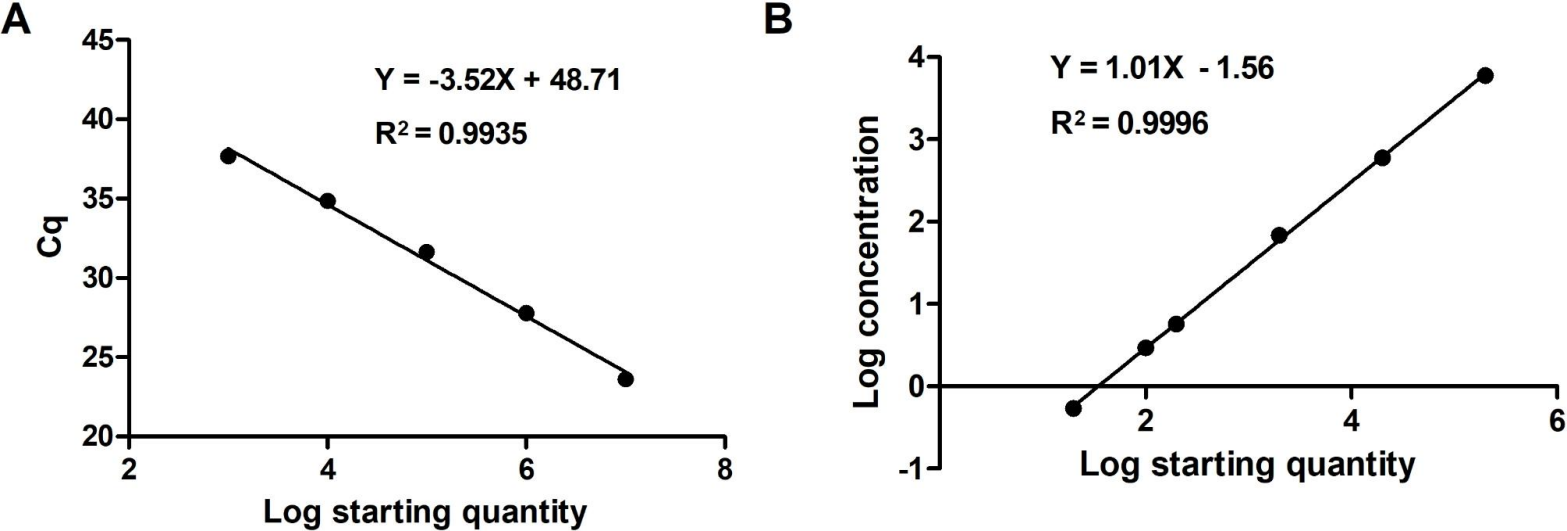
Quantification of serially diluted porcine circovirus 4 plasmid by ddPCR and qPCR. (**A**) Standard curves of pSK-PCV4 plasmid constructed by qPCR. The quantification correlation was obtained by plotting the quantification cycle value against the log calculated pSK-PCV4 concentration in dilutions. (**B**) Standard curves by ddPCR. The quantification correlation was obtained by plotting the log absolute concentration against the log calculated pSK-PCV4 concentration in dilutions

Assays with serially diluted pSK-PCV4 plasmid solution exhibited good linearity in both qPCR and ddPCR. In qPCR, the standard curve exhibited a good linear correlation (Y = − 3.52X + 48.71) with R^2^ value of 0.9935 (Fig. 3A), the detection limit was 5.71 copies/µL (Table 1). In contrast, the standard curve of the ddPCR assay was Y = 1.01x − 1.56 with R^2^ value of 0.9996 (Fig. 3B), the LoD was 0.54 copies/µL (Table 1). The results revealed that the LoD of ddPCR was ~ 10.6-fold lower than that of qPCR, which indicated that ddPCR was significantly more sensitive for PCV4 detection. In the repeatability tests, the intra-assay coefficient of variation ranged from 1.22 to 3.70%, and the coefficient of variation of the inter-assay ranged from 2.79 to 7.57% (Table 2). These results showed that the developed PCV4 ddPCR has a good reproducibility.

**Table 1:** Comparison of quantitative real-time PCR and droplet digital PCR using serially diluted PCV4 plasmid

Calculated concentration of pSK-PCV4 in dilution (copies/µL)	qPCR (mean Cq value)	ddPCR (mean concentration, copies/µL)
2.0 × 10^6^	23.61	Overload
2.0 × 10^5^	27.79	6002.8
2.0 × 10^4^	31.63	596.6
2.0 × 10^3^	34.87	68.6
2.0 × 10^2^	37.67	5.71
1.0 × 10^2^	ND	2.95
2.0 × 10^1^	ND	0.54
2.0 × 10^0^	ND	ND
NTC	ND	ND

Cq = quantification cycle; NTC = no template control; ND = not detected

**Table 2:** Robustness and reproducibility analysis of droplet digital PCR

Calculated concentration of pSK-PCV4 in dilution (copies/µL)	Intra-assay variation (robustness)			Inter-assay variation (reproducibility)		
	Mean (copies/µL)	SD	CV (%)	Mean (copies/µL)	SD	CV (%)
2.0 × 10^5^	6002.8	73.1	1.22	5862.8	163.7	2.79
2.0 × 10^4^	596.6	13.3	2.23	578.4	21.0	3.63
2.0 × 10^3^	68.6	1.97	2.87	65.7	3.93	5.98
2.0 × 10^2^	5.71	0.19	3.31	5.37	0.38	7.01
1.0 × 10^2^	2.95	0.07	2.49	2.65	0.18	6.60
2.0 × 10^1^	0.54	0.02	3.70	0.45	0.04	7.57

CV = coefficient of variation; SD = standard deviation

### Analytical specificity of the ddPCR assay

**Fig. 4:**
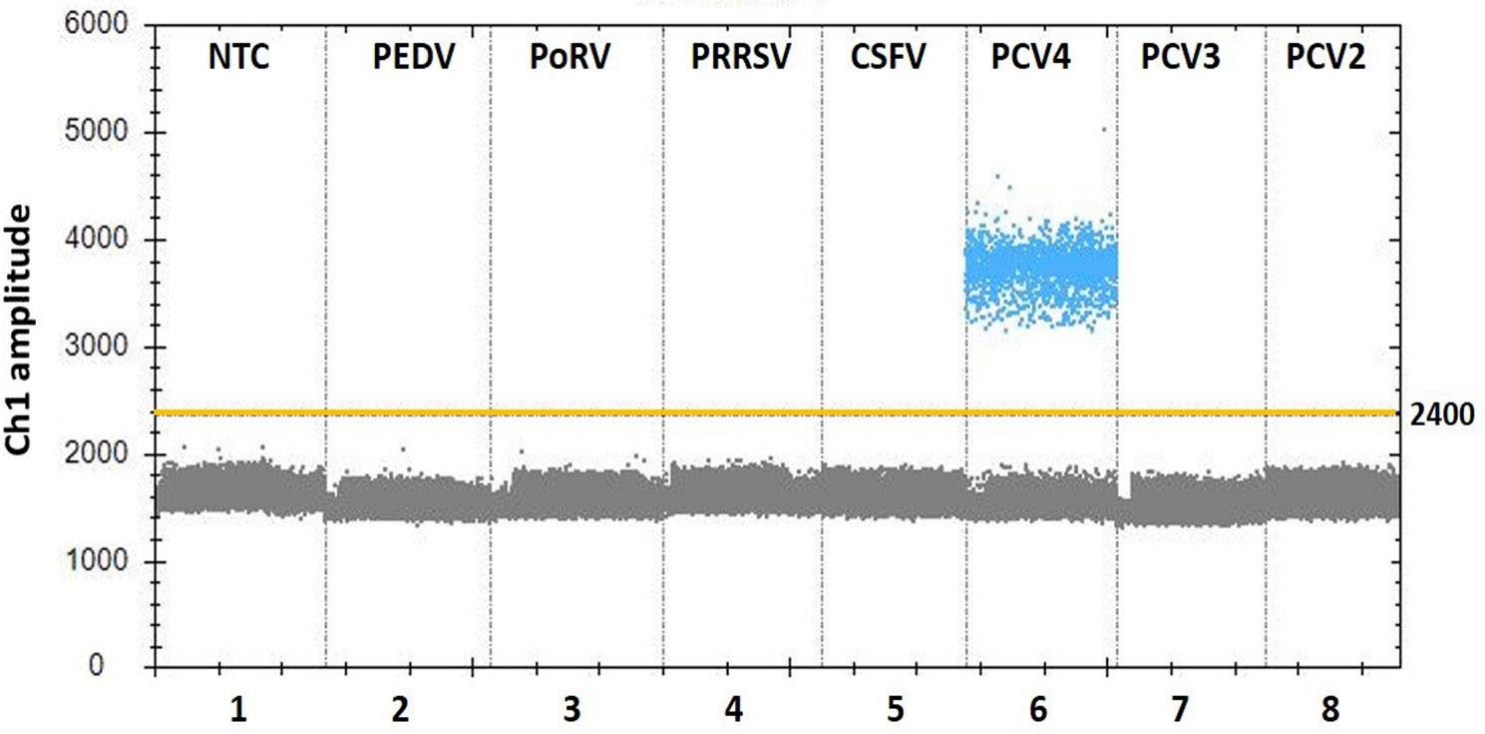
Specificity analysis of the PCV4 ddPCR assay. Lanes 1 to 8 (divided by vertical black dotted lines): the fluorescence amplitude of NTC, PEDV, PoRV, PRRSV, CSFV, PCV4, PCV3, and PCV2, respectively. The manually set threshold for droplet positivity is represented by the yellow horizontal line

For the specificity analysis, nucleic acid templates from different pathogens were assayed, including PCV4, PCV3, PCV2, PEDV, PoRV, CSFV and PRRSV. As shown in Fig. 4, only the PCV4 test was positive, while other pathogen tests were negative. The results indicated that this method exhibits specificity for the detection of PCV4.

### Clinical samples testing

To further determine if ddPCR can be performed in a routine involving real world subjects, 160 clinical samples collected from 24 pig farms in Henan Province were evaluated using ddPCR and qPCR. As shown in Table 3, PCV4 was detected with a positive rate of 4.4% (7 of 160) by ddPCR and 5.6% (9 of 160) by qPCR. Two samples detected as negative by qPCR were positive by ddPCR. The amplicons from samples giving conflicting positive results were further sequenced by Sangon Biotech Co. Ltd. (Beijing, China), and the sequencing results confirmed that the two samples were positive for PCV4. According to these data, ddPCR was found to be more sensitive than qPCR for PCV4 detection in clinical samples.

**Table 3:** Comparison of ddPCR and qPCR sensitivity for PCV4 clinical samples

qPCR	ddPCR		Total
	Positive	Negative	
Positive	7	0	7
Negative	2	151	153
Total	9	151	160

## Discussion

Since its discovery in 2019, PCV4 has been detected in pigs of all ages and in both clinically healthy and on diseased pigs [21–23]. A latest study showed that PCV4 was pathogenic to piglets after challenge with the virus generated from infectious clones [24], indicating that PCV4 may pose a potential threat to the pig industry. To date, PCV4 has not been isolated from clinical samples, which severely hinders the in-depth research of the epidemiology and pathogenic mechanism of the virus infection. To monitor PCV4 continuously and effectively, several etiological and serological methods have been developed and played an important role in the diagnosis of PCV4 infection. However, these methods are time-consuming, complex to operate, and unsuitable for samples with low virus load [25]. Therefore, it is urgently needed to develop a rapid, simple and sensitive detection method for PCV4.

The ddPCR is emerging as an attractive platform that enables absolute quantification of nucleic acid targets without relying on the establishment of a standard curve as required in qPCR. The ddPCR technology does not depend on a standard curve and the reaction is efficient and highly sensitive, thus it has been used to detect a variety of diseases and is especially useful for low viral load samples [16, 17, 26]. In addition, ddPCR is highly tolerant to many PCR inhibitors, making it more suitable for the detection of complex clinical samples such as blood and faeces [27]. Because of these features of high sensitivity, absolute quantification and high reproducibility, ddPCR has been widely used for viral load quantification [28, 29], mutant genes detection [30], target verification following genome editing [31], copy number variations analysis [32], etc.

In this study, a sensitive and specific ddPCR method for detection and quantification of PCV4 was successfully established. Meanwhile, a qPCR assay, which used the same primers and probe as ddPCR was also developed to cross-validate both assays. To evaluate linearity, sensitivity, and repeatability of ddPCR and qPCR, serially diluted pSK-PCV4 plasmid solution were prepared in triplicate, and then used to conduct parallel tests. The results indicated that both ddPCR and qPCR exhibited good linearity, with R^2^ values of 0.9996 and 0.9935, respectively. The LoD of ddPCR and qPCR were 0.54 copies/µL and 5.71 copies/µL, respectively. Indicating that the sensitivity of the ddPCR assay was 10.6 times higher than that of qPCR, which is consistent with the findings from PCV2 and PCV3 ddPCR assay [18, 19]. Plasmid standard dilutions with different copy numbers were also used to evaluate the robustness and reproducibility of the ddPCR assay. The results showed that the intra- and inter-assay coefficients of variation (CV) for concentration (copies/µL) were 1.22 to 3.70% and 2.79 to 7.57%, respectively, which indicated that the robustness and repeatability of the ddPCR reaction system was high. Additionally, the ddPCR assay exhibited high specificity, presenting no cross-reactivity signals with other common swine pathogens such as PCV3, PCV2, PEDV, PRRSV, CSFV and PoRV. These advantages make the PCV4 ddPCR assay more suitable for the early detection of PCV4 infection.

Subsequently, the method was used for the detection of PCV4 in clinical samples to evaluate the practicability of ddPCR and qPCR. The qPCR-positive detection rate was 4.4%, while PCV4 ddPCR exhibited a greater positive (5.6%). Different results were obtained from ddPCR and qPCR, indicating that the sensitivity of the ddPCR method was higher than that of qPCR. In addition, all the positive samples were collected from the pigs without any symptoms, indicating that PCV4 could cause subclinical infection and cofactors may be essential for the virulence of PCV4. Thus, ddPCR is a specific, sensitive and rapid-detection method of high clinical significance for the early infection diagnosis and a potential epidemic tracking tool for PCV4 in pig farms.

Despite the advantages described above, the utility of the ddPCR assay is limited by several factors. First, the ddPCR technology is not widely available in veterinary clinical diagnosis due to its high cost. Second, the ddPCR method started to lose linearity when the initial concentration of the nucleic acid templates was higher than 1 × 10^5^ copies/µL, thereby presenting a relatively narrow linear dynamic range compared with the qPCR assay [33, 34]. Third, the controls used in the ddPCR assay might be not precise representative of the target template, leading to the underestimation of the true sample concentration. In conclusion, further efforts are necessary to develop more accurate and standardized approaches for improving the ddPCR assays.

## Conclusion

We first established and evaluated a droplet digital PCR assay for rapid and accurate detection of PCV4. PCV4 ddPCR exhibits higher sensitivity compared with qPCR, and it was analytically specific and reproducible, making it a reliable tool for the diagnosis and epidemiological investigation of PCV4.

### Electronic supplementary material

Below is the link to the electronic supplementary material.

**Table S1** Cq values (qPCR) and target copy number (ddPCR) of PCV4 positive clinical samples.

## Data Availability

The data presented in this manuscript are available through the corresponding author upon reasonable request. The datasets generated and/or analysed during the current study are available in the GenBank repository, accession numbers: PEDV strain LYL: ON960076; PoRV strain CC0812: JF835112; PCV2 strain SH: HM038027; PCV3: MZ449239.

## Abbreviations

PCV4: Porcine circovirus 4
ddPCR: Droplet digital PCR
qPCR: Real-time quantitative PCR
PCVAD: Porcine circovirus-associated disease
PDNS: Porcine dermatitis and nephropathy syndrome
PCV2: Porcine circovirus type 2
PCV3: Porcine circovirus type 3
PoRV: Porcine rotaviru
PEDV: Porcine epidemic diarrhoea virus
CSFV: Classical swine fever virus
PRRSV: Porcine reproductive and respiratory syndrome virus
LoD: Detection limit
