# Supplementary material for: Development of a droplet digital PCR method for detection of porcine circovirus 4
Source: BMC Vet Res. 2023 Aug 22;19:129. doi: 10.1186/s12917-023-03690-5 (PMC10464377; doi:10.1186/s12917-023-03690-5)
Supplement: Supplementary file 1 — Table S1 Cq values (qPCR) and target copy number (ddPCR) of PCV4 positive clinical samples. [file 12917_2023_3690_MOESM1_ESM.docx]

**Table S1 Cq values (qPCR) and target copy number (ddPCR) of PCV4 positive clinical samples**

| Sample number | Sample type | Number of replicates | ddPCR | |  | qPCR | |
| --- | --- | --- | --- | --- | --- | --- | --- |
|  |  |  | Copy number/μL | Mean^a^ ± SD^b^ |  | Cq values | Mean ± SD |
| 1 | Blood | 3 | 73 | 74±2 |  | ND^c^ | ND |
|  |  |  | 76 |  |  | ND |  |
|  |  |  | 72 |  |  | ND |  |
| 2 | Blood | 3 | 1053 | 1057±5 |  | 32.5 | 32.63±0.23 |
|  |  |  | 1062 |  |  | 32.9 |  |
|  |  |  | 1055 |  |  | 32.5 |  |
| 3 | Blood | 3 | 836 | 844±8 |  | 33.8 | 34.43±0.57 |
|  |  |  | 852 |  |  | 34.9 |  |
|  |  |  | 845 |  |  | 34.6 |  |
| 4 | Blood | 3 | 273 | 265±7 |  | 38.4 | 39.63±0.32 |
|  |  |  | 262 |  |  | 39.0 |  |
|  |  |  | 260 |  |  | 38.5 |  |
| 5 | Blood | 3 | 958 | 952±14 |  | 33.8 | 33.50±0.30 |
|  |  |  | 961 |  |  | 33.5 |  |
|  |  |  | 937 |  |  | 33.2 |  |
| 6 | Blood | 3 | 472 | 473±13 |  | 36.5 | 36.63±0.15 |
|  |  |  | 460 |  |  | 36.8 |  |
|  |  |  | 486 |  |  | 36.6 |  |
| 7 | Blood | 3 | 27 | 29±4 |  | ND | ND |
|  |  |  | 26 |  |  | ND |  |
|  |  |  | 33 |  |  | ND |  |
| 8 | Inguinal lymph node | 3 | 1013 | 1009±18 |  | 32.7 | 32.93±0.25 |
|  |  |  | 1014 |  |  | 32.9 |  |
|  |  |  | 989 |  |  | 33.2 |  |
| 9 | Kidney | 3 | 1372 | 1348±28 |  | 31.2 | 31.13±0.21 |
|  |  |  | 1355 |  |  | 31.3 |  |
|  |  |  | 1317 |  |  | 30.9 |  |

^a^Mean: the average of the copy numbers per reaction from three independent ddPCR reactions.

^b^SD: standard deviation. ^c^ND: not detected.
